# Supplementary material for: Comparative Transcriptomic Analysis Revealed Potential Differential Mechanisms of Grass Carp Reovirus Pathogenicity
Source: Int J Mol Sci. 2023 Oct 24;24(21):15501. doi: 10.3390/ijms242115501 (PMC10649309; doi:10.3390/ijms242115501)
Supplement: Supplementary file 1 [file ijms-24-15501-s001.zip › ijms-2656757-supplementary.pdf]

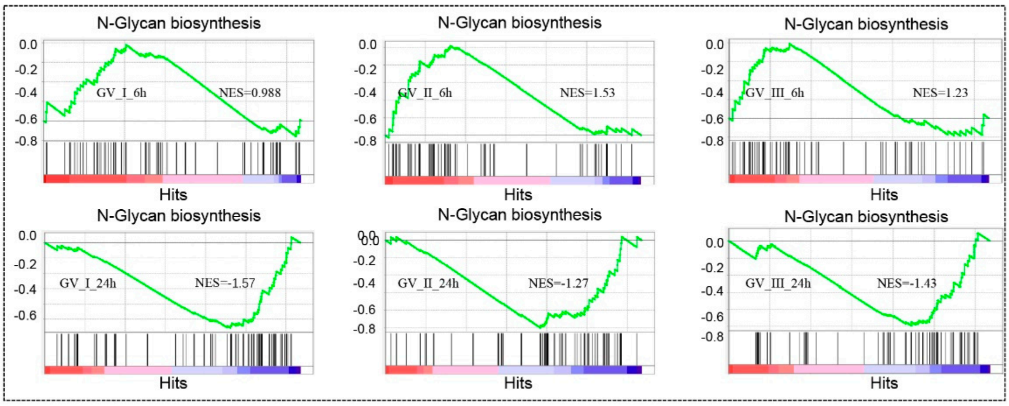

Figure S1. GSEA was conducted to analyze the DEGs in CIK cells at 6 and 24 h with three different types of GCRV. The DEGs exhibited a noteworthy enriched in N-Glycan biosynthesis at 6 h, and down-regulated at 24 h.

Table S1. Summary statistics for sequence quality control and mapped data of samples.

Sample	Raw reads	Raw bases	Clean reads	Clean bases	Error rate (%)	Q30 (%)	GC content（%）	Total mapped	Multiple mapped	Uniquely mapped
Con6h1	49,694,168	7,006,570,244	49,693,944	7,005,597,914	0.043	94.85	46.46	97.21%	4.16%	93.05%
Con6h2	50,189,336	7,017,057,653	50,189,118	7,015,862,514	0.0402	96.12	46.85	97.2%	3.77%	93.43%
Con6h3	49,644,584	6,932,430,269	49,644,376	6,931,256,185	0.0399	96.23	47.29	97.12%	3.66%	93.46%
Con24h1	50,151,692	6,984,714,619	50,151,500	6,983,591,639	0.0397	96.4	47.2	97.04%	3.71%	93.33%
Con24h2	50,114,000	6,989,163,929	50,113,766	6,987,962,270	0.0404	96.03	47.29	97.01%	3.75%	93.26%
Con24h3	49,649,240	6,907,524,358	49,649,016	6,906,405,472	0.0409	95.94	47.35	97.04%	3.72%	93.32%
GVI6h1	49,760,394	7,002,543,988	49,760,212	7,001,483,343	0.0399	96.28	47.21	96.98%	3.80%	93.18%
GVI6h2	50,030,486	6,976,862,433	50,030,266	6,975,724,026	0.0399	96.3	47.67	96.91%	4.64%	92.27%
GVI6h3	49,427,718	6,984,073,130	49,427,536	6,982,983,862	0.0402	96.11	47.39	96.98%	3.97%	93.01%
GVI24h1	48,988,518	6,922,436,698	48,988,334	6,921,461,568	0.0398	96.33	47.27	97.13%	4.08%	93.05%
GVI24h2	49,223,430	6,922,306,650	49,223,206	6,921,218,602	0.0403	96.04	47.3	96.26%	3.82%	92.44%
GVI24h3	49,149,496	6,914,378,502	49,149,266	6,913,360,904	0.0405	96.07	47.47	97.06%	4.11%	92.95%
GVII6h1	49,468,568	6,935,080,297	49,468,354	6,934,124,811	0.0436	94.59	47.8	97.25%	3.78%	93.46%
GVII6h2	49,141,346	6,927,738,839	49,141,138	6,926,608,800	0.0404	95.97	47.69	97.18%	3.67%	93.51%
GVII6h3	50,785,578	7,048,624,489	50,785,286	7,047,395,535	0.0398	96.36	47.43	97.35%	3.70%	93.65%
GVII24h1	50,222,768	7,003,380,166	50,222,578	7,002,268,597	0.0395	96.49	47.04	97.12%	3.40%	93.73%
GVII24h2	49,983,240	6,990,394,547	49,983,058	6,989,211,081	0.0407	95.87	47.05	97.07%	3.67%	93.40%
GVII24h3	49,659,936	6,906,215,920	49,659,794	6,905,138,868	0.0407	96.02	47.3	97.01%	3.59%	93.42%
GVIII6h1	49,773,336	7,006,371,848	49,773,152	7,005,413,652	0.0445	94.05	47.1	97.05%	3.56%	93.50%
GVIII6h2	49,933,952	6,989,754,312	49,933,712	6,988,595,825	0.0405	95.96	46.37	97.23%	3.54%	93.69%
GVIII6h3	48,674,750	6,867,316,099	48,674,498	6,866,187,997	0.0407	95.78	46.37	97.28%	3.81%	93.46%
GVIII24h1	49,888,732	7,001,881,334	49,888,548	7,000,896,152	0.0419	95.44	47.33	96.94%	3.72%	93.22%
GVIII24h2	50,454,838	7,060,951,530	50,454,662	7,059,868,370	0.0402	96.16	46.95	97.07%	3.77%	93.30%
GVIII24h3	49,695,968	6,929,450,152	49,695,792	6,928,360,561	0.0417	95.54	47.17	97.02%	3.72%	93.30%
